# The Therapeutic Potential of Glucagon-like Peptide 1 Receptor Agonists in Traumatic Brain Injury

**DOI:** 10.3390/ph17101313

**Published:** 2024-10-01

**Authors:** Anja Harej Hrkać, Kristina Pilipović, Andrej Belančić, Lea Juretić, Dinko Vitezić, Jasenka Mršić-Pelčić

**Affiliations:** 1 Department of Basic and Clinical Pharmacology and Toxicology, Faculty of Medicine, University of Rijeka, 51000 Rijeka, Croatia; anja.harej.hrkac@medri.uniri.hr (A.H.H.); andrej.belancic@medri.uniri.hr (A.B.); lea.juretic@medri.uniri.hr (L.J.); dinko.vitezic@medri.uniri.hr (D.V.); jasenka.mrsic.pelcic@medri.uniri.hr (J.M.-P.); 2 Department of Clinical Pharmacology, Clinical Hospital Centre Rijeka, 51000 Rijeka, Croatia

**Keywords:** brain injuries, traumatic, glucagon-like peptide 1, incretins, neuroprotection

## Abstract

Traumatic brain injury (TBI), which is a global public health concern, can take various forms, from mild concussions to blast injuries, and each damage type has a particular mechanism of progression. However, TBI is a condition with complex pathophysiology and heterogenous clinical presentation, which makes it difficult to model for in vitro and in vivo studies and obtain relevant results that can easily be translated to the clinical setting. Accordingly, the pharmacological options for TBI management are still scarce. Since a wide spectrum of processes, such as glucose homeostasis, food intake, body temperature regulation, stress response, neuroprotection, and memory, were demonstrated to be modulated after delivering glucagon-like peptide 1 (GLP-1) or GLP-1 receptor agonists into the brain, we aimed to speculate on their potential role in TBI management by comprehensively overviewing the preclinical and clinical body of evidence. Based on promising preclinical data, GLP-1 receptor agonists hold the potential to extend beyond metabolic disorders and address unmet needs in neuroprotection and recovery after TBI, but also other types of central nervous system injuries such as the spinal cord injury or cerebral ischemia. This overview can lay the basis for tailoring new research hypotheses for future in vitro and in vivo models in TBI settings. However, large-scale clinical trials are crucial to confirm their safety and efficacy in these new therapeutic applications.

## 1. Introduction

An increasing body of evidence indicates that employing incretin-based therapy might be a promising neuroprotective strategy in the treatment of various central nervous system (CNS) conditions, such as the brain ischemia, as well as neurodegenerative disorders, e.g., Parkinson’s and Alzheimer’s disease [1,2,3,4,5,6]. The neurotrophic and neuroprotective effects exerted by glucagon-like peptide 1 (GLP-1) receptor agonists and glucose-dependent insulinotropic polypeptide (GIP) mimetics have been studied in non-clinical and clinical settings, and it has been found, in addition to their metabolic effects, that they have neuroprotective and neurotrophic properties in the injured CNS [7,8,9,10,11,12]. They exert these effects directly within the CNS by reducing oxidative stress, decreasing apoptosis, diminishing neuroinflammation, improving mitochondrial function, and promoting neuronal survival [13,14,15].

The exploration of incretin-based treatments for traumatic brain injury (TBI) is still in its early stages, with most studies conducted in preclinical settings, as one of the main challenges is translating the promising results from animal studies into successful clinical outcomes [11]. The main hurdles in research efforts are the complexity of TBI in humans, including the variability in injury severity and patient responses, as well as determining the optimal timing for administration and the appropriate dosage of incretin-based therapies.

In this review, the aim is to present a case for the use of GLP-1 receptor agonist (GLP-1RA)-based therapeutic strategies in the treatment of TBI, including its long-term consequences, as well as adjacent conditions, by providing a summary of the current results from preclinical and clinical studies.

## 2. Search Strategy

The articles considered in this narrative review related to TBI and GLP-1RAs were electronically retrieved from the PubMed database using the following search terms: brain injuries, traumatic; liraglutide; exenatide; incretins; glucagon-like peptide 1. We considered articles that were published from 2015 to 2024. The identified articles were screened by title and abstract, and articles that were inconsistent with our inclusion criteria were excluded from further analysis.

To deduce, search strategy on semaglutide and tirezepatide (which is a GLP-1 RA/GIP agent) was also subsequently performed. However, at the present time, the results are limited only to those articles relevant for diabetes, obesity, and cardiovascular outcomes fields. To the best of our knowledge, there are no studies on semaglutide nor tirzepatide relevant for inclusion in this review. However, there is a high certainty that the results we described on “older” GLP-1RAs (e.g., liraglutide, and exenatide) can be extrapolated—because the mechanisms we are describing are mostly interconnected with the primary mechanism of the group.

## 3. Epidemiology and the Consequences of Traumatic Brain Injury

As one of the major causes of death and disability worldwide, TBI is tied to significant socio-economic consequences and high medical costs and is, thus, a substantial burden for both TBI patients and society. The estimates say that 69 million individuals experience TBI each year worldwide. The most common causes of TBI are falls and traffic-related accidents [16], with others being violence, sports-related, accidents in the home or at work, and suicides or suicide attempts [17].

In terms of gender and age distribution, males are more commonly affected by TBI in younger age groups. This could be attributed to the predominant causes of brain trauma in this demographic, such as traffic accidents, violent activities, and sports injuries, where younger men are often involved. However, the scenario changes in the elderly population, where TBI becomes a more prevalent cause of injury with a more balanced distribution between males and females, as the cause of brain trauma is more commonly falls.

Clinically, TBI is classified as mild, moderate, or severe, based on the initial severity scoring, i.e., Glasgow coma scale (GCS). Mild brain trauma (also known as concussion) is the most common TBI diagnosis with 81%, followed by moderate with 11%, and severe TBI that is found in 8% of all cases. Other than GCS, valuable tools to evaluate the clinical severity of TBI are the duration of altered levels of consciousness (LOC), post-traumatic amnesia (PTA), as well as neuroimaging findings (Table 1).

Aside from its immediate consequences, brain trauma also contributes to increased mortality in the long term. Studies have indicated that individuals who have experienced TBI are twice as likely to die within the first year after the injury compared to individuals of similar age, gender, and race who have not experienced such an injury. Furthermore, TBI has been shown to decrease life expectancy by an average of 7 years [18].

TBI has been linked to various neurodegenerative diseases including Alzheimer’s and Parkinson’s diseases, amyotrophic lateral sclerosis (ALS), frontotemporal dementia (FTD), and chronic traumatic encephalopathy (CTE) [19,20]. Additionally, it increases the risk of neuroendocrine disorders such as hypopituitarism and psychiatric disorders such as obsessive–compulsive disorder, anxiety, mood disorders, addiction, as well as the incidence of suicide. Moreover, TBI can lead to non-neurological issues such as sexual dysfunction, bladder/bowel incontinence, musculoskeletal problems, fractures, and metabolic dysfunctions affecting amino acid metabolism, exacerbating neurological symptoms [21].

## 4. Pathophysiology of Traumatic Brain Injury

Pathological processes in brain trauma, triggered by mechanical damage, can be classified as the primary and the secondary injury. The primary injury, as the immediate result of head impact, causes direct damage to neural tissue. Examples of primary TBI-induced lesions are contusions, diffuse axonal injury, concussion, hematomas, and penetrating injuries. The latter can occur when an object (such as a bullet, knife, or the bone fragment) penetrates the skull and damages the brain tissue directly. These primary injuries can lead to a cascade of secondary injuries, which may occur in the minutes, hours, or days following the initial trauma.

Secondary injury after TBI can be divided into processes that can be directly linked to the primary damage and those that develop in the aftermath of brain trauma. Initially, TBI causes necrotic cell death and tearing and shearing of brain tissue, which results in neuronal, axonal, and vascular damage.

One of the first events following neuronal injury is the rapid release of excitatory amino acids (EAAs), mainly glutamate and aspartate from the presynaptic nerve endings, with concomitant failure of the EAAs’ uptake into cells, mainly astrocytes, due to the dysfunction of specific transporters [22,23,24]. Through the activation of ionotropic glutamate receptors, such as the ligand-gated ion channels’ receptors N-methyl-d-aspartate (NMDA) receptor and α-amino-3-hydroxy-5-methyl-4-isoxazole propionate (AMPA) receptor, EAAs cause cell membrane depolarization followed by the influx of cations, such as sodium (Na^+^), calcium (Ca^2+^), and potassium (K^+^), into the cells [25]. It is especially an increase in Ca^2+^ concentrations that are linked to the activation of enzymes, e.g., phospholipases, calpains, and endonucleases, that can affect cell integrity by altering membranes, the cytoskeleton, and the DNA integrity, resulting in cell damage and death. Glutamate also binds to metabotropic receptors, which regulate Ca^2+^ and downstream signaling via GTP-binding proteins, cause the activation of the phospholipase C/inositol-1,4,5-triphosphate system, and mobilize the release of additional Ca^2+^ from the intracellular storage.

The increase in intracellular Ca^2+^ results in intense reactive oxygen species (ROS) and reactive nitrogen species (RNS) production, predominantly due to the impaired mitochondrial electron transport chain and the activation of the calcium dependent enzymes, proteases, and phospholipases [26]. The accumulation of Ca^2+^ in the cytosol is additionally responsible for the activation of a number of proteins connected to the apoptotic cell death propagation, including calcineurin, calpain, and caspases.

Inflammation is one of the important processes involved in the cascade of secondary events resulting from TBI. The results of previously conducted studies indicate possible harmful, but also neuroprotective, effects of inflammation, and therefore, its role in the pathophysiology of TBI is still insufficiently known. Following brain trauma, in the earlier post-injury stages, dysfunction of the blood–brain barrier (BBB) allows the infiltration of peripheral, blood-derived cells such as neutrophils, monocytes, and lymphocytes into the injured brain parenchyma [27]. In addition, post-TBI neuroinflammation can be triggered by the presence of tissue and cellular debris, the release of cytokines and chemokines, the increased synthesis of prostaglandins, as well as ROS and RNS [28]. In the CNS, traumatic injury activates the resident immune response cells, namely microglia, astrocytes, and oligodendrocytes. Microglial activation in the injured brain is necessary for the removal of cell debris, a process that is needed for the attenuation of inflammation and promotion of tissue repair. However, microglia themselves are responsible for the release of neurotoxic substances that might contribute to neuronal damage and death. Although traditionally considered as support cells for neurons, astrocytes also play a crucial role in the immune response of the CNS. They help maintain the BBB, regulate neurotransmitter levels, modulate inflammation, and contribute to the release of neurotrophic factors promoting tissue repair. They can also contribute to the formation of a glial scar following injury, which helps protect undamaged tissue but may also inhibit regeneration [29,30,31].

Among pathological processes involved in the progression of TBI-related neurodegenerative processes, disturbances of metabolism play a very significant role. Predominant metabolic perturbation is a disturbance in the glucose metabolism, and hyperglycemia was found to be one of the most common TBI complications. Namely, in the brain, glucose is the main source of energy through its metabolism to lactate and pyruvate, as the brain cells tend not to use the beta-oxidation of fatty acids for energy production [32]. Subsequently, any perturbation between energy demands and the levels of glucose in the brain might become a cause of further pathobiological cascades. Studies suggest that the cause of disturbance in the brain glucose metabolism after brain trauma is insulin resistance [33,34]. However, this diabetogenic state is also characterized by the upregulation of amino acid metabolism, ketoacidosis, and an increase in lipogenesis [35].

## 5. Current State of Traumatic Brain Injury Management

Understanding and managing both primary and secondary injuries are critical in the treatment and rehabilitation of TBI patients. As the primary injuries are inevitable and cannot be treated after they occur, the only avenue to affect them is by preventative measures. Conversely, there is a great possibility to modify different elements related to the secondary injury processes. At this time, in the clinical setting, options for the management of brain trauma patients are limited almost exclusively to symptomatic treatments, as there is no therapeutic approach proven to be effective in modifying the post-TBI secondary injury [36,37]. Currently, TBI treatment is based on the severity of the injury. In mild TBI cases, usually no treatment is required, except advising the affected to rest and use over-the-counter pain relief medications for the headache, which is common in these patients. These individuals are usually not hospitalized but need to be monitored at home in case of worsening of the condition or the occurrence of new symptoms. In the case of moderate to severe TBI, measures related to basic and advanced life support need to be taken. These include proper oxygenation, with intubation, if necessary, maintenance of blood pressure and volume, and measures related to the normalizing partial pressure of carbon dioxide (pCO₂). One of the main goals is to regulate the cerebral blood flow (CBF) and not to allow it to go under the so-called ischemic threshold, which is considered to be at CBF less than 20 mL/100 g/min. When it comes to defective CBF regulation, it may occur early after trauma or develop later, and it can be transient or persistent; however, this happens irrespective of the TBI severity. CBF is directly dependent on cerebral perfusion pressure (CPP), and it is more significant to maintain adequate CPP vs. intracranial pressure (ICP); although, it is also ICP that can contribute to CBF reduction. These measurements related to the intensive care of TBI patients, if not done correctly, can additionally contribute to secondary damage [38].

As for the neuroprotective strategies in the TBI management, they have been thus far based on particular secondary post-injury processes. However, even though numerous preclinical studies with very limited success in translation to clinical setting. There are a number of potential causes of failure of these studies: limitations regarding the therapeutic time window, heterogenicity of the traumatic injuries suffered by humans in relation to controlled preclinical settings, or the presence of secondary insults, such as ischemia, infection, hypovolemia, and polytrauma.

It has been suggested, based on evidence from various studies, that one of possible strategies in TBI management is the use of drugs or drug combinations that target multiple secondary injury mechanisms [39,40,41]. This includes drugs with effects that stimulate the recovery and optimal functioning not only of neuronal, but also non-neuronal cells. In addition, new therapeutic strategies need to consider that TBI does not only impact the brain, but also has systemic effects that need to be addressed in brain trauma patients. In that regard, incretin-system-based therapies might be promising for their multifunctional and multiorgan effects.

## 6. The Position of GLP-1RAs in Diabetes, Obesity, and Other Metabolic Fields

The discovery of GLP-1RAs represents a monumental success in medical science, offering a powerful tool to combat the surging global epidemics of obesity, expected to increase from 650 million to 2 billion by 2035, and type 2 diabetes, projected to rise from 500 million to 1.3 billion by 2050 [42].

GLP-1RAs lower glucose by maintaining a therapeutic level of GLP-1, which leads to increased insulin secretion, decreased glucagon production, and delayed stomach emptying. These medications are classified as short-acting (exenatide, lixisenatide) or long-acting (liraglutide, semaglutide, dulaglutide, exenatide extended release, albiglutide) and are predominantly licensed for subcutaneous injections; with the exception of semaglutide (Rybelsus), which is the peroral agent [43,44,45]. GLP-1RAs have been demonstrated to lower HbA1c levels by at least 0.8–1.6%. GLP-1RA treatment has significant glucose-lowering effects, anti-obesogenic properties, minimal risk of hypoglycemia, and minor adverse events (mostly gastrointestinal, such as nausea, vomiting, and diarrhea) [44]. Furthermore, liraglutide, dulaglutide, and semaglutide also demonstrated benefits in three-component major adverse cardiovascular event outcomes (MACE-3: cardiovascular death, nonfatal myocardial infarction, or nonfatal stroke) in LEADER (Liraglutide Effect and Action in Diabetes: Evaluation of Cardiovascular Outcome Results), REWIND (Researching Cardiovascular Events With a Weekly Incretin in Diabetes), and SUSTAIN-6 (Semaglutide Unabated Sustainability in Treatment of Type 2 Diabetes) cardiovascular outcome trials, respectively [46,47,48]. It is important to highlight that liraglutide, dulaglutide, and semaglutide provide considerable cardiovascular benefits and are licensed for usage in patients with severe forms of renal impairment (eGFR ≥ 15). Very recently, a FLOW trial was published, and subcutaneous semaglutide also demonstrated a reduction in the risk of clinically important kidney outcomes and cardiovascular death in patients with diabetes mellitus type 2 and chronic kidney disease [49].

Preclinical studies indicate that GLP-1RAs reduce body weight by directly interacting with several GLP-1R populations and influencing neuronal circuits related to food intake, reward, and energy expenditure [50]. Clinical research consistently shows that using long-acting GLP-1RA leads to considerable weight loss, likely because of altered dietary preferences and reduced energy intake. Currently, two GLP-1RA (liraglutide 3 mg/day s.c. and semaglutide 2.4 mg/week s.c.) medications and one GLP-1RA/GIP ‘twincretin’ (tirzepatide 5–15 mg/week s.c.) are indicated for the treatment of a non-syndromic obesity as adjuncts to lifestyle modifications [51]. They demonstrated a similar safety profile as in diabetes trials and a mean weight loss (after 56–72 weeks) of −6.4%, −15.8%, and −22.5%, respectively [52,53,54].

Reductions in body weight, liver damage indicators, and liver fat content have all been seen with GLP-1 receptor agonists. There is evidence to support the notion that these medications can slow the progression of hepatic fibrosis and help a non-negligible percentage of NASH patients resolve their steatohepatitis. As things stand, there is no proof that GLP-1 RAs can help individuals with NAFLD who already have liver fibrosis. However, the information following long-term GLP-1 RA treatment is still lacking [55].

Last but not least, aside from being essential glucose-lowering and anti-obesity drugs, GLP-1RAs appear to have promising anti-inflammatory, lung protective properties and a positive impact on gut microbiome composition, which Belančić et al. have already extensively overviewed elsewhere [43].

It is also worth mentioning that GLP-1RAs administration is association with a low risk of hypoglycemia, as well as non-serious adverse events only (predominantly gastrointestinal and transitory, e.g., nausea, vomiting, diarrhea). GLP-1RA administration is not recommended in cases of acute pancreatitis development, presence of gastroparesis, and a family history of (medullary) thyroid cancer [43].

An increasing body of evidence points to the possible benefits of the use of incretin-based therapeutic approaches in combating various non-metabolic conditions [56,57]. This is particularly true in the research of the effects of these drugs within the central and peripheral nervous system, as it has been proven that incretin mimetics possess neuroprotective and neurotrophic actions, in in vitro and in rodent models, offering protection against neurodegenerative conditions, such as ischemia, stroke, Alzheimer’s disease, Parkinson’s disease, and TBI.

GLP-1 and GLP-1 receptors in the brain are involved in a wide range of physiological functions, including the regulation of appetite, energy balance, neuroprotection, cognitive function, reward processing, stress response, cardiovascular regulation, and glucose homeostasis. These roles highlight the importance of GLP-1 signaling in maintaining overall brain health and function, with implications for both metabolic and neurological diseases [57,58,59,60,61].

Specifically, in the CNS, GLP-1 is synthesized by pre-proglucagon neurons in the nucleus tractus solitarius and functions as a neurotransmitter, helping to regulate feeding behavior and maintaining intracellular homeostasis [62]. GLP-1 receptors were detected in several brain regions, including the hypothalamus, hippocampus, and cortex, all of which are heavily involved in cognitive function [63].

## 7. Molecular Mechanisms Underlying the Potential Neuroprotective Actions of GLP-1 Receptor Agonists in CNS Injuries

Although preclinical studies consistently show the neuroprotective effects of GLP-1 agonists, the exact mechanisms remain unclear. Upon release into the circulation, two incretin hormones, GLP-1 and GIP, modify glucose clearance by stimulating postprandial insulin secretion [64]. This is mediated by two types of G-protein-coupled receptors (GPCRs) that are activated upon the incretins, both of which are predominantly coupled to the G_αs_ subunit, which activates adenylyl cyclase to increase intracellular Camp [59]. In addition, other signaling pathways are activated, such as the mobilization of intracellular calcium as a significant signaling pathway.

In 1984, Hoosein and Gurd discovered that GLP-1 activates signaling in the brain, proposing its role as a neurotransmitter and/or neuroendocrine effector [65]. However, our understanding of GLP-1R-mediated intracellular signaling within the CNS remains limited. Research indicates that GLP-1R in various brain regions operates through the G_αs_ protein, activating protein kinase A and increasing calcium influx. This activation also stimulates mitogen-activated protein kinases, triggers the PI3K/AKT/mTOR pathway, and enhances glutamatergic transmission via protein-kinase-A-mediated phosphorylation of the AMPA receptor. However, some studies suggest that GLP-1RAs may exert their effects through mechanisms beyond GLP-1 receptors, potentially involving unknown receptors or pathways. Additionally, research has revealed regional differences in cell signaling pathway involvement, underscoring the need for further studies to elucidate the mechanisms of GLP-1 action.

GLP-1 receptor agonisms have been shown to affect different mechanisms of the secondary injury, not only in TBI, but also other conditions that share the same pathophysiological processes, notable examples being cerebral ischemia (CI), epilepsy, and various neurodegenerative disorders. These mechanisms include excitotoxicity, oxidative stress, inflammation, and apoptosis.

Romano et al. [66] found that exendin-4, GLP-1RA which shares 53% structural homology with GLP-1, downregulated the gene expression of subunits 2A and 2B of the glutamate receptor NMDA in the hippocampus, simultaneously increasing the phosphorylation of the NMDA receptor 2B subunit. Moreover, it is believed that GLP-1 receptors have key roles on glutamatergic, but not GABAergic, neurons [67]. GLP-1RAs increase the expression and activity of several antioxidant enzymes, such as superoxide dismutase, glutathione peroxidase, and catalase, and additionally activate the CREB-BDNF-TrkB signaling pathway, which further stimulates the production of antioxidant proteins. They were found to inhibit the activity of NADPH oxidase, particularly NOX4, a major source of ROS in endothelial cells, and GLP-1RAs activate the nuclear-factor-erythroid-2-related factor 2 (Nrf2), a master regulator of antioxidant responses.

Neuroinflammation is the inflammatory response within the CNS, often associated with neurodegenerative diseases, where it contributes to neuronal damage and exacerbates disease progression. GLP-1 is involved with multiple anti-inflammatory pathways that potentially can be used to diminish neuroinflammation, providing neuroprotection. Treatments targeting the GLP-1 pathway provide reduced inflammation-induced synaptic impairments, reduced glial activation, decreased inflammatory molecules’ expression, and inhibited inflammatory signaling pathways [68,69,70].

Altered BBB function plays a crucial role in neurotoxicity in conditions such as the ischemic and hemorrhagic stroke or in brain trauma. Astrocytes, key components of the BBB, contribute to its breakdown by secreting inflammatory factors. The GLP-1 receptor, expressed on astrocytes, can be targeted by GLP-1RAs, protect the BBB, and reduce brain inflammation. Different studies demonstrate that astrocytes may have different phenotypes, which can exert neurotoxic or neuroprotective effects, and that targeting the GLP-1 receptor disrupts the astrocytes’ transformation towards the neurotoxic A1 phenotype [70].

Antiapoptotic effects of GLP-1RAs in the CNS pathologies have been detected in different experimental studies [7,71,72]. The mechanisms of these effects are proposed to connect with the activation of the pro-survival PI3K/AKT and MAPK pathways and influence the ratio of anti-apoptotic vs. apoptotic proteins, e.g., Bcl-2/Bax balance, with subsequent effects on the key executioner of apoptosis, caspase-3, preventing the cascade of events that lead to programmed cell death.

Notably, modified GLP-1RAs, engineered for more effective receptor activation, exhibit greater neuroprotective efficacy than those currently in clinical use [4]. Although many mechanisms have been presented, the definite mechanisms by which GLP-1 and GLP-1RAs play a neuroprotective role have not been fully elucidated.

## 8. GLP-1 Receptor Agonists in Traumatic Brain Injury

Incretin mimetics represent a promising class of drugs in the treatment of mTBI, which is based on the use of the endogenous incretin signaling system GLP-1, GIP, glucagon (Gcg), and their receptors [11,73]. These drugs are interesting not only because they have extra-pancreatic effects, especially in the central and peripheral nervous system, but also due to the fact that they have emerged with neurotrophic and neuroprotective actions in both in vivo and in vitro models of neurodegenerative disorders. The determination of a synergistic GLP-1/GIP or GLP-1/Gcg effect has led to the development of dual GLP-1/GIP and GLP-1/Gcg receptor coagonists with the potential for enhanced efficacy than either mimetic alone [74]. Unimolecular combinations of GIP, GLP-1, and Gcg have been shown to be a more physiologically balanced and amenable incretin combination for the diverse needs of human patients and show promise in several models of neurodegeneration. GLP-1-based polypharmacologic approaches to the treatment of metabolic diseases, and possibly neurological disorders, seem to hold much promise, as single therapeutics often have limited effectiveness.

Why are incretin mimetics interesting for the potential therapy of neurological disorders? Their ability to decrease neuroinflammation, excitotoxicity, oxidative stress, and apoptosis in animal models of a wide range of neurological disorders, including autoimmune encephalomyelitis, retinal neurodegeneration, stroke, and ALS of these processes, is also implicated in the progress of secondary TBI [44].

It has been proven that β-cells treated with exendin-4 before the induction of oxidative stress by the addition of hydrogen peroxide resulted in a 41.7% decrease in apoptosis by inhibiting the c-Jun N-terminal kinase and glycogen-synthase-kinase-3β-mediated apoptosis pathway through AKT. Resulting from pre-treatment with exendin-4 was also the activation of caspase-9 and -3 [11].

Further evidence for the antioxidant effects of incretin-based therapies comes from the use of the DPP-IV inhibitor sitagliptin in a mouse model of TBI [75], which resulted in increased manganese superoxide dismutase (MnSOD) production and overall improved outcomes.

Another important neuroprotective effect of incretin-based therapies includes a strong anti-inflammatory component, namely the reduction of glial cell activation and related cytokine production [11]. In models of TBI and related in vitro studies, incretin-based therapies have shown efficacy through increased CREB signaling and anti-inflammation [75], including reduced glial cell activation and cytokine production, using the independent administration of GIP and the GLP-1 analogue slow-release exenatide PT-302.

In another study, using chinchillas as animals and repeated blast exposures as a model mild traumatic brain injury (mTBI), the protective role of liraglutide as a glucagon-like peptide-1 receptor agonist was investigated, focusing on hearing damage of the animals caused by repeated blast exposures. The research found that liraglutide treatment, administered either before or after blast exposure, significantly mitigated hearing loss and auditory damage. This suggests that liraglutide has potential neuroprotective properties that could be beneficial in preventing or reducing hearing impairment related to mTBI [76].

Another study was carried out to investigate the effect of liraglutide on neuronal cultures and a mouse model of mTBI. It highlights the neurotrophic and neuroprotective properties of liraglutide in cultures—SH-SY5Y cells and SH-SY5Y cells overexpressing GLP-1 receptors—and a mouse model of mTBI. Liraglutide stimulated dose-dependent proliferation in SH-SY5Y cells and a GLP-1R overexpressing cell line at low concentrations. Pre-treatment with liraglutide effectively protected neuronal cells from cell death induced by oxidative stress and glutamate excitotoxicity. The neuroprotective effects, comparable to those of liraglutide, are likely mediated through the cAMP/PKA/pCREB pathway. In a mouse model of mTBI, liraglutide administered post-injury at clinically relevant doses improved memory function at both 7 and 30 days post-trauma, supporting its therapeutic potential for mTBI [77].

GLP-1RA exendin-4 was approved for the treatment of type 2 diabetes mellitus and also demonstrated a neurotrophic effect in different neurological disorders. The neuroprotective effects of exendin-4 were confirmed on neurological outcomes, cerebral blood flow, neurodegeneration, and inflammatory responses using a cortical contusion impact (CCI) injury model in rats [78]. TBI in rats led to neurological impairments, neurodegeneration, reduced cerebral blood flow, and increased inflammatory responses, and exendin-4 treatment facilitated the recovery of neurological and cognitive functions, improved cerebral blood flow, and reduced both neural degeneration and inflammatory cytokine levels following TBI. Additionally, exendin-4 significantly decreased TBI-induced overexpression of TNFα and IL-1β, as well as phosphorylation of p38 and ERK1/2. These results suggest that exendin-4, has strong therapeutic potential for TBI by improving neurological outcomes through the attenuation of inflammatory responses [9].

There is a study that provides the pharmacokinetic properties and effectiveness of clinically available sustained-release exenatide formulation (SR-Exenatide) PT302 in a concussive mTBI mouse model. PT302 was administered subcutaneously (s.c.) in different dosages, and plasma concentrations of the drug were measured in different time points over three weeks. There was an observed initial rapid release of exenatide into the plasma, followed by a secondary phase of sustained release, both in a dose-dependent manner. Visual and spatial deficits caused by weight drop-induced mTBI were mitigated by a single post-injury treatment with exenatide delivered through the s.c. injection of PT302 at clinically relevant doses, both in the short-term (7 days) and longer-term (30 days) protocols. The neurotrophic and neuroprotective effects of slow-release exenatide were confirmed by immunohistochemical analysis in the mTBI mouse model [79].

There is some evidence supported in previous research that oxidative stress and mitochondrial disfunction have an impact on the neuronal damage after TBI. Two drugs that are known for their antioxidant effects are L-carnitine and exendin-4. A study was carried out for the examination of antioxidant properties and to improve neurological function in rats with moderate TBI. Two drugs were given to the rats immediately after the injury for 15 days; after that, a neurological function examination and brain histology were performed. Both treatments showed improved sensory and motor functions, oxidative stress was decreased, and mitochondrial ROS content was reduced. Additionally, only the treatment with exendin-4 improved memory in treated rats [80].

Synaptophysin is one of the main integral transmembrane proteins of synaptic vesicles, and it is also a molecular marker for the synapse and a functional brain marker [81,82]. Mild blast traumatic brain injury (B-TBI) leads to lasting cognitive impairments, particularly in novel object recognition, with milder effects on mice Y-maze behavior. B-TBI also decreases synaptophysin (SYP) protein levels in cortical and hippocampal tissues. Administration of exendin-4 via subcutaneous pumps to the mice either 48 h before or 2 h after B-TBI prevented cognitive deficits and SYP changes.

The neuronal cell line HT22 was utilized in a study in which the cells were subjected to biaxial stretch injury in vitro. Cells were treated with exendin-4 before injury; exendin-4 pre-treatment (25 to 100 nM) attenuated the cytotoxic effects of biaxial stretch damage and preserved neurite length similar to sham treated cells [81].

B-TBI-induced neurodegeneration, cognitive changes, and the expression of genes associated with dementia disorders were investigated in a study in which the treatment with exendin-4 was given either pre- or post-injury. The protective effect on B-TBI-induced neurodegeneration was determined at 72 h, memory deficits at days 7–14, and diminished expression of genes modified by the blast at day 14 post-injury [83].

Recently, one synthetic dual incretin receptor agonist, herein referred to as “twincretin,” enhanced the metabolic benefits of single receptor agonists in mouse and monkey models with type 2 diabetes. Twincretin is an injectable form of tirzepatide, a novel GLP-1 and GIP agonist for the treatment of type 2 diabetes [84]. The neuroprotective effects of twincretin were investigated in the cell and mouse models of mTBI. It increased intermediates in the neurotrophic CREB pathway and enhanced the viability of human neuroblastoma cells exposed to toxic concentrations of glutamate and hydrogen peroxide, insults that model the inflammatory conditions in the brain following mTBI. Twincretin was also shown to enhance the neurotrophic activity of single incretin receptor agonists in the mentioned cells. A clinically translatable dose of twincretin, upon administration post-mTBI, is observed to completely reinstate the visual and spatial memory deficits induced by mTBI, as assessed in a mouse model. These results suggest twincretin as a new neuroprotective agent, and it may achieve additional benefits beyond those of single incretin receptor agonists through dual agonism [85].

Overview of the studies using the GLP-1 receptor agonists in in vitro and in vivo TBI models are provided in Table 2. Despite conducting a comprehensive and careful review of the currently available literature, we were unable to identify any published studies that have specifically investigated the use of GLP-1RAs in TBI in clinical settings. Furthermore, there appears to be no ongoing or completed clinical trials that explore this particular treatment paradigm. This highlights a notable gap in the existing research, underscoring the need for future studies to evaluate the clinical applications of GLP-1RAs.

## 9. GLP-1 Receptor Agonists in Various Central Nervous System Conditions

### 9.1. GLP-1 Receptor Modulation in Cerebral Ischemia

CI is a leading cause of death and disability globally, imposing significant public health and social burdens. CI causes damage to multiple cells in the brain and blood vessels including neurons and glial and vascular endothelial cells. The increased permeability of capillaries and BBB observed in the ischemic core, the penumbra, and other areas is leading to perivascular and perineuronal edema. Ischemia progression involves various pathological processes, such as inflammation, excitotoxicity, oxidative stress, neuronal loss, glial activation, and BBB dysfunction. In addition to ischemic injury itself, the reperfusion process (restoring cerebral perfusion after CI) has also been shown to cause damage to brain cells [87].

CI is a condition with limited medical treatment options and its therapy primarily aims to restore cerebral blood flow and reduce neurological impairment. Although current advances in recanalization therapies have improved recovery from ischemic stroke, developing neuroprotective therapies remains essential to safeguard the brain during and before reperfusion, extend the intervention window, and enhance outcomes [88]. Over the past few decades, many promising drugs have not been successfully translated into clinical practice, indicating the significant need to develop new neuroprotective treatment strategies [89].

GLP-1RAs have shown promising neuroprotective effects in preclinical studies of stroke and demonstrated the potential of GLP-1 to reduce acute ischemic damage in the brain.

GLP-1RAs were extensively studied in animal models of brain ischemia. A comprehensive review from 2022 examined over 45 studies involving mouse and rat models, with the literature reviewed spanning from January 2011 to August 2021 [4]. These studies investigated the effects of various GLP-1RAs in both diabetic and non-diabetic animals. The findings generally have indicated reductions in infarct volume, neurological deficits, and oxidative stress markers as well as increased survival rates following treatment with GLP-1 RAs. Different GLP-1RAs have been studied in CI models, including exenatide, liraglutide, lixisenatide, and semaglutide, and have reported promising results. It is important to emphasize that GLP-1RAs demonstrated a neuroprotective effect without affecting the blood glucose levels. There is strong evidence from animal experiments that GLP-1 and GLP-1RAs are neuroprotective in stroke, and these findings are very convincing, because they have been replicated in a different laboratory and by using different animal models of stroke, with or without diabetes or hyperglycemia.

It is, however, necessary to continue further investigations to test the neuroprotective mechanisms of GLP-1 and GLP-1RAs. In the future, large-scale clinical trials are the necessary procedure to verify the results revealed in animal experiments and to guarantee their clinical application to patients suffering stroke. The indications, safety, efficacy, and mechanisms of action of GLP-1RAs in CI patients will be the focus of clinical trials [4].

### 9.2. Potential of the Use of GLP-1 Receptor Agonists in the Spinal Cord Injury

Spinal cord injury (SCI) is a severe neurological condition that leads to significant motor, sensory, and autonomic dysfunctions, causing lifelong physical and mental distress. Despite various therapeutic interventions, a definitive cure remains elusive due to the complex mechanisms involved [90].

Recent studies have highlighted the potential of GLP-1RAs in neuroprotection and recovery, offering new opportunities for SCI treatment.

SCI involves a primary mechanical injury followed by secondary biochemical and vascular consequences, exacerbating the initial damage, very similar to TBI pathophysiology but with certain specificities related to this specific CNS region. The pathophysiology of SCI includes acute and chronic phases, characterized by ischemia, oxidative stress, inflammation, apoptosis, and neurodegeneration [91].

Current therapeutic approaches aim to reduce secondary damage and promote neuronal recovery through neuroprotective and neuroregenerative strategies. Despite varying degrees of success, a definitive cure remains elusive due to the complex mechanisms involved in healing and protection [92].

Preclinical studies have demonstrated the potential of GLP-1RAs in SCI models. GLP-1 receptor activation has been associated with reduced neuroinflammation, enhanced axonal regeneration, and improved functional recovery [93]. For example, the administration of sitagliptin, a DPP4 inhibitor, in a rat model of SCI increased GLP-1R protein levels, reduced neuronal apoptosis, and promoted axon regeneration [94]. Similarly, exenatide, a GLP-1RA, has shown promising results in mitigating endoplasmic reticulum stress and improving functional outcomes post-SCI [95]. Additionally, GLP-1RAs, such as exendin-4, have demonstrated neuroprotective effects by inhibiting the mitochondrial apoptotic pathway and promoting macrophage polarization towards the M2 phenotype, which is associated with anti-inflammatory responses [96].

The neuroprotective and metabolic regulatory properties of GLP-1RAs highlight their potential in clinical applications for SCI treatment. For instance, dulaglutide and semaglutide have shown efficacy in reducing nonfatal strokes and major adverse cardiovascular events, which can indirectly benefit SCI patients by improving overall cardiovascular health and reducing secondary complications [97]. Future research should focus on elucidating the precise mechanisms of GLP-1-receptor-mediated neuroprotection in SCI. This includes investigating the roles of different GLP-1RAs, optimizing dosing regimens, and exploring combinatory therapies to enhance therapeutic outcomes. Additionally, long-term studies are necessary to evaluate the sustained effects of GLP-1 receptor activation on neuronal recovery and functional improvement in SCI patients. Further investigation is also needed to understand the potential of GLP-1RAs in mitigating cognitive impairment and peripheral neuropathy, extending their application beyond diabetes and major cardiovascular events [88].

## 10. Conclusions

The discovery and clinical implementation of GLP-1RAs have revolutionized the treatment of diabetes and obesity, two conditions projected to reach epidemic proportions globally. GLP-1RAs not only provide significant glucose-lowering effects but also offer substantial benefits in terms of weight reduction, cardiovascular protection, and renal safety. Beyond their established roles in diabetes and obesity management, emerging evidence suggests that GLP-1RAs possess extensive neuroprotective properties, offering potential therapeutic benefits in various CNS disorders, including TBI, ischemic stroke, SCI, and neurodegenerative diseases such as Alzheimer’s and Parkinson’s. The mechanisms underlying these effects include the modulation of neuroinflammation, oxidative stress, excitotoxicity, and apoptosis, as well as the enhancement of neurogenesis and axonal regeneration. Given the promising preclinical and clinical data, the use of GLP-1RAs might be expanded beyond metabolic disorders, potentially addressing significant unmet needs in neuroprotection and recovery in CNS injuries and diseases. However, further research is necessary to fully elucidate the mechanisms of GLP-1-mediated neuroprotection and to optimize the clinical use of GLP-1RAs in these indications. Large-scale clinical trials are crucial to confirm the efficacy and safety of GLP-1RAs in these new therapeutic areas and to explore their full potential in improving neurological outcomes and overall patient health.

To summarize, incretin-based therapies offer a variety of beneficial signaling end points that can easily be applied to TBI pathophysiological consequences. Within the neurotrophic/neuroprotective, antioxidant, and anti-inflammatory properties that underlie the actions of incretin-based therapies, all of which show beneficial effects on TBI pathophysiology, these compounds represent potential therapy for treatment of TBI consequences. Since the effects of even one TBI injury can be long-term, there is a great and immediate need for an effective drug therapy for the treatment of secondary injuries related to TBI. Designer biased, incretin-receptor agonists are now being developed, and additional research is needed to refine such drugs to neurological disorders. It is, therefore, reassuring to note that many FDA-approved incretin-based therapies are entering into clinical trials for neurological diseases by using the dose and route of administration approved and shown to be effective in treating type 2 diabetes. What is called for is a concerted effort in applying these safe and efficacious drugs to trials for the treatment of TBI.

## Figures and Tables

**Table 1:** Assessment of TBI severity according to the patient’s clinical presentation and structural imaging.

	Glasgow Coma Scale (GCS)	Duration of Loss of Consciousness (LOC)	Post-Traumatic Amnesia (PTA)	Neuroimaging
Severity	A numerical assessment tool utilized to evaluate a patient’s consciousness and neurological status following a brain injury. Scoring is based on the best eye-opening response (1–4 points), motor response (1–6 points), and verbal response (1–5 points).	The length of time a person remains unconscious following TBI.	The period following a brain injury during which a patient experiences a gap in their memory, not being able to form continuous memories of events occurring around them.	Computerized tomography (CT) is frequently primary imaging modality in the acute phase, but magnetic resonance imaging (MRI) has better sensitivity and specificity, particularly in the identification of diffusion axonal injury.
Mild	13–15	<20 min to 1 h	A few mins to less than 24 h	Normal
Moderate	9–12	1 h to 24 h	1–7 days	Normal or abnormal
Severe	3–8	>24 h	>7 days	Abnormal

**Table 2:** Overview of the studies using the GLP-1 receptor agonists in in vitro and in vivo traumatic brain injury models.

GLP-1 Receptor Agonist	Model	Outcomes	References
In Vitro Studies			
Exendin-4	Oxidative stress in β cells	Decrease in apoptosis	Kim et al. [86]
Slow-release exenatide PT-302	Glial cells	Anti-inflammation	Bader et al. [79]
Liraglutide	SH-SY5Y cells and SH-SY5Y cells overexpressing GLP-1 receptors	Dose-dependent proliferation in SH-SY5Y cells and a GLP-1R overexpressing cell line, pre-treatment effectively protected neuronal cells from cell death induced by oxidative stress and glutamate excitotoxicity	Li et al. [77]
Exendin-4	SH-SY5Y cells, primary neuron cultures, rodent primary cerebral cortical neurons	In neuronal cultures, exendin-4 ameliorated H_2_O_2_-induced oxidative stress and glutamate toxicity	Rachmany et al. [78]
Exendin-4	HT22 cells (neuronal-derived cell line)	Attenuated cytotoxic effect of biaxial stretch damage, preserved neurite length, protected against neurite shrinkage and cell-death-maintained neurite length	Rachmany et al. [81]
Twincretin	SH-SY5Y cells	Increased levels of intermediates in the neurotrophic CREB pathway and enhanced viability of human neuroblastoma cells exposed to toxic concentrations of glutamate and hydrogen peroxide	Tamargo et al. [85]
**In Vivo Studies**			
Sitagliptin	mTBI in mouse model	Increased manganese superoxide dismutase (MnSOD) production and overall improved outcomes	DellaValle et al. [75]
Liraglutide	mTBI in chinchillas	Mitigated hearing loss and auditory damage	Jiang et al. [76]
Liraglutide	mTBI in mouse model	Improved memory function	Li et al. [77]
Exendin-4	mTBI in mouse model	Fully ameliorated mTBI-induced deficits in novel object recognition	Rachmany et al. [81]
Exendin-4	CCI in rats	Recovery of neurological and cognitive functions, improved cerebral blood flow, reduced both neural degeneration and inflammatory cytokine levels significantly diminished the TBI-induced overexpression of TNFα and IL-1β	Zhang et al. [9]
Exenatide	mTBI in mouse model	Mitigation of short- and longer-term cognitive impairments (visual and spatial deficits)	Bader et al. [79]
Exendin-4 L-carnitine	mTBI in rat model	Improved sensory and motor functions, improved memory, oxidative stress decrease ameliorated	Chen et al. [80]
Exendin-4	B-TBI in mouse model	Neurodegeneration and memory deficits	Tweedie et al. [83]
Exendin-4	B-TBI in mouse model	Prevented cognitive deficits	Rachmany et al. [81]
Twincretin	mTBI in mouse model	Restored the visual and spatial memory deficits induced by mTBI	Tamargo et al. [85]

Abbreviations: mTBI, mild traumatic brain injury; CCI, controlled cortical impact; B-TBI, blast traumatic brain injury.

## Data Availability

No new data were created or analyzed in this study. Data sharing is not applicable to this article.
